# Supplementary material for: Biohybrid Tendons Enhance the Power‐to‐Weight Ratio and Modularity of Muscle‐Powered Robots
Source: Adv Sci (Weinh). 2025 Nov 30;13(15):e12680. doi: 10.1002/advs.202512680 (PMC13042981; doi:10.1002/advs.202512680)
Supplement: Supplementary file 1 — Supporting Information [file ADVS-13-e12680-s002.pdf]

Supporting Information

Biohybrid tendons enhance the power-to-weight ratio and modularity of muscle-powered robots

*Nicolas Castro,[1] Ronald Heisser, [1] Maheera Bawa,[1] Bastien Aymon,[1] Sarah Wu,[1] Annika Marschner,[1] Sonika Kohli,[1] Angel Bu,[1] Laura Rosado,[1] Martin Culpepper,[1] Xuanhe Zhao,[1] Ritu Raman[1]**

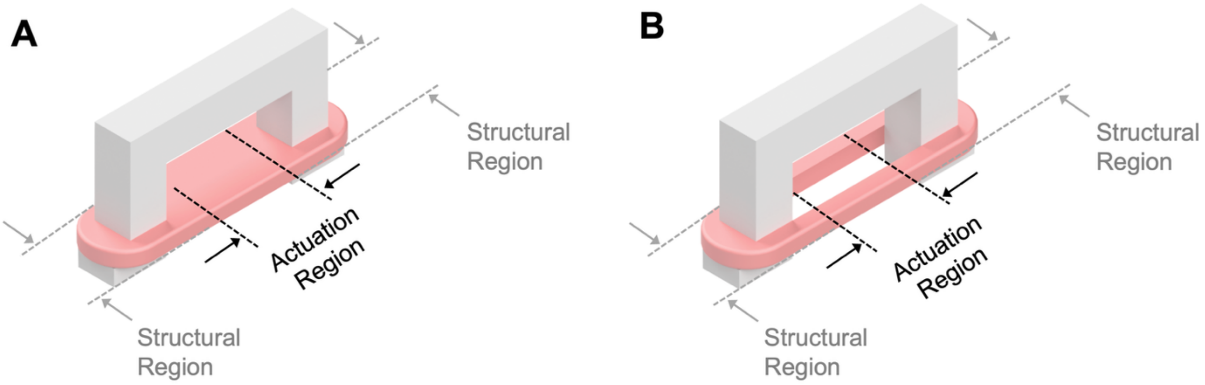

Figure S1. Schematic of typical muscle strip (top) and ring (bottom) designs typically used for biohybrid robots. A large percentage of the tissue is typically used to form a robust connection with the skeleton and thus not used for actuation.

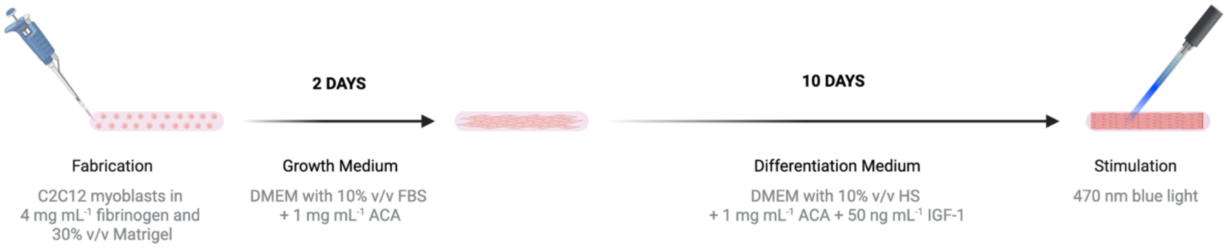

Figure S2. Schematic of muscle fabrication. C2C12 myoblasts are embedded in a fibrin/Matrigel hydrogel, maintained in growth medium for 2 days, and differentiated for 10 days prior to MTU fabrication and testing.

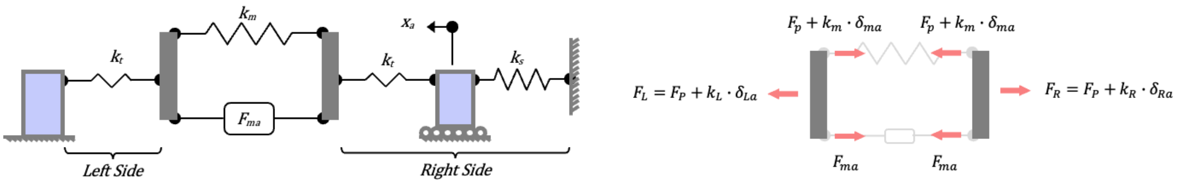

STIFFNESS DEFINITIONS

STIFFNESS OF ELEMENTS LEFT OF THE MUSCLE

$k_L = k_t$

STIFFNESS OF ELEMENTS RIGHT OF THE MUSCLE

$$k_R = \left(\frac{1}{k_t} + \frac{1}{k_s}\right)^{-1} = \frac{k_t \cdot k_s}{k_t + k_s}$$

k_t = *Tendon stiffness*
k_s = *Skeleton stiffness*
k_L = *Stiffness of elements left of muscle*
k_R = *Stiffness of elements right of muscle*

COMPATIBILITY DURING ACTUATION

ELONGATIONS OF ELEMENTS

$\delta_{La} + \delta_{ma} + \delta_{Ra} = 0$; *Elongations sum to 0 as they're chained between fixed ends*

ELONGATIONS OF ELEMENTS RIGHT OF MUSCLE

$$\delta_{Ra} = \delta_{ta} + \delta_{sa} = \delta_{ta} + x_a$$

δ_{La} = *Elongation of elements left of muscle*
δ_{Ra} = *Elongation of elements right of muscle*
δ_{ma} = *Muscle elongation due to actuation*
δ_{ta} = *Tendon elongation due to actuation*
δ_{sa} = *Skeleton elongation due to actuation*
x_a = *Pin displacement due to actuation*

STATICS DURING ACTUATION

SUM OF FORCES ON RIGHT AND LEFT OF MUSCLE WHILE MUSCLE IS ACTUATED

$$F_L = F_R \rightarrow F_P + k_L \cdot \delta_{La} = F_P + k_R \cdot \delta_{Ra} \rightarrow k_L \cdot \delta_{La} = k_R \cdot \delta_{Ra} \rightarrow \delta_{La} = \delta_{Ra} \cdot \frac{k_R}{k_L}$$

F_L = *Force left of muscle*
F_R = *Force right of muscle*
F_P = *Preload force*

COMPATIBILITY + STATICS DURING ACTUATION

ACTUATED ELONGATION OF MUSCLE AND ELEMENTS RIGHT OF MUSCLE

$$\delta_{La} + \delta_{Ra} = -\delta_{ma} \rightarrow \delta_{Ra} \cdot \frac{k_R}{k_L} + \delta_{Ra} = -\delta_{ma} = \delta_{Ra}\left(\frac{k_R}{k_L} + 1\right) \rightarrow -\delta_{ma} = \delta_{Ra}\left(\frac{k_R + k_L}{k_L}\right)$$

TENDON ELONGATION AND PIN DISPLACEMENT DURING ACTUATION

$$F_{ta} = F_{sa} \rightarrow k_t \cdot \delta_{ta} = k_s \cdot \delta_{sa} \rightarrow \delta_{ta} = \delta_{sa} \cdot \frac{k_s}{k_t} \rightarrow \delta_{ta} = x_a \cdot \frac{k_s}{k_t}$$

F_{ta} = *Force increment in tendon due to actuation*
F_{sa} = *Force increment in skeleton due to actuation*

SUM OF FORCES AT RIGHT MUSCLE-TENDON INTERFACE

$$F_P + k_m \cdot \delta_{ma} + F_{ma} = F_P + k_R \cdot \delta_{Ra} \rightarrow F_{ma} = F_P + k_R \cdot \delta_{Ra} - F_P - k_m \cdot \delta_{ma} \rightarrow F_{ma} = k_R \cdot \delta_{Ra} + k_m \cdot (-\delta_{ma})$$

Substitute: $-\delta_{ma} = \delta_{Ra}\left(\frac{k_R + k_L}{k_L}\right)$

$$F_{ma} = k_R \cdot \delta_{Ra} + k_m \cdot \delta_{Ra} \cdot \left(\frac{k_R + k_L}{k_L}\right) = \delta_{Ra} \cdot \left(k_m \cdot \left(\frac{k_R + k_L}{k_L}\right) + k_R\right) = \delta_{Ra} \cdot \left(\frac{k_m}{k_L} \cdot k_R + k_m + k_R\right)$$

Substitute: $\delta_{Ra} = \delta_{ta} + x_a$

$$F_{ma} = (\delta_{ta} + x_a) \cdot \left(\frac{k_m}{k_L} \cdot k_R + k_m + k_R\right)$$

Substitute: $\delta_{ta} = x_a \cdot \frac{k_s}{k_t}$

$$F_{ma} = \left(x_a \cdot \frac{k_s}{k_t} + x_a\right) \cdot \left(\frac{k_m}{k_L} \cdot k_R + k_m + k_R\right) = x_a \cdot \left(\frac{k_s + k_t}{k_t}\right) \cdot \left(\frac{k_m}{k_L} \cdot k_R + k_m + k_R\right)$$

$$F_{ma} = x_a \cdot \left(\frac{k_s + k_t}{k_t}\right) \cdot \left(\frac{k_m}{k_L} \cdot k_R + k_m + k_R\right)$$

Substitute: $k_L = k_t$ *Substitute:* $k_R = \frac{k_t \cdot k_s}{k_t + k_s}$

$$F_{ma} = x_a \cdot \left(\frac{k_s + k_t}{k_t}\right) \cdot \left(\frac{k_m}{k_t} \cdot \frac{k_t \cdot k_s}{k_t + k_s} + k_m + \frac{k_t \cdot k_s}{k_t + k_s}\right)$$

$$F_{ma} = x_a \cdot \left[\left(\frac{k_s + k_t}{k_t}\right) \cdot \frac{k_m}{k_t} \cdot \frac{k_t \cdot k_s}{k_t + k_s} + \left(\frac{k_s + k_t}{k_t}\right) \cdot k_m + \left(\frac{k_s + k_t}{k_t}\right) \cdot \frac{k_t \cdot k_s}{k_t + k_s}\right] = x_a \cdot \left[\frac{k_m \cdot k_s}{k_t} + k_s \cdot \frac{k_m}{k_t} + k_t \cdot \frac{k_m}{k_t} + k_s\right] = x_a \cdot \left[2 \cdot \frac{k_m \cdot k_s}{k_t} + k_m + k_s\right]$$

MUSCLE FORCE EQUATIONS

$$\boldsymbol{F_{ma} = x_a \cdot \left[2 \cdot \frac{k_m \cdot k_s}{k_t} + k_m + k_s\right]}$$ ***INTERNAL MUSCLE FORCE***

$$\boldsymbol{F_{me} = k_s \cdot x_a}$$ ***EXERTED MUSCLE FORCE TRANSMITTED TO SKELETON***

$$\boldsymbol{\frac{F_{me}}{F_{ma}} = \frac{k_s}{k_m + 2 \cdot \frac{k_m \cdot k_s}{k_t} + k_s}}$$ ***RATIO OF TRANSMITTED MUSCLE FORCE TO INTERNAL MUSCLE FORCE***

Figure S3. Derivation of muscle force equations. Modeling the muscle, tendons, and skeleton as linear elastic springs enables deriving an expression for muscle internal actuation force and external transmitted force as a function of muscle, tendon, and skeleton stiffnesses.

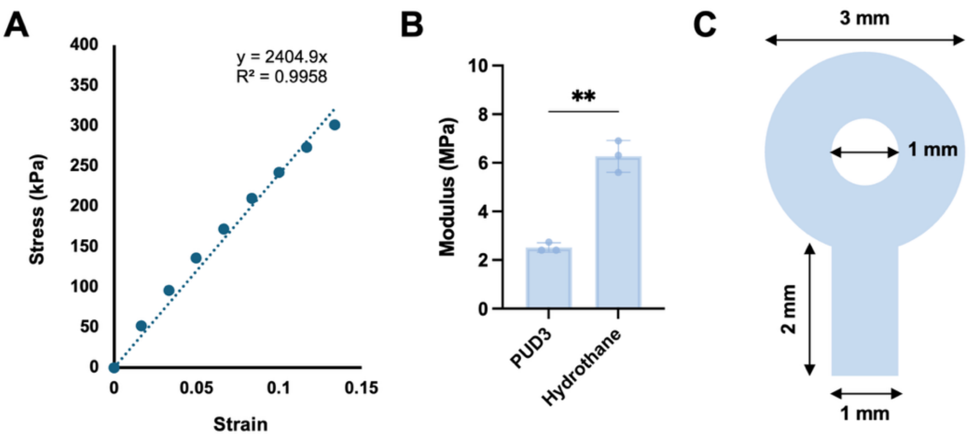

Figure S4. Tendon characterization. (**A**) Representative data from tensile testing of a PVA:PAA hydrogel tendon with Polyurethane D3 (PUD3) backing. (**B**) Elastic modulus for tendons with PUD3 and Hydrothane backings (n = 3 per group, Welch's t-test, ** $p < 0.01$). Error bar indicates standard deviation. (**C**) Dimensions of tendons used in this study. Tendons were 0.6 mm thick.

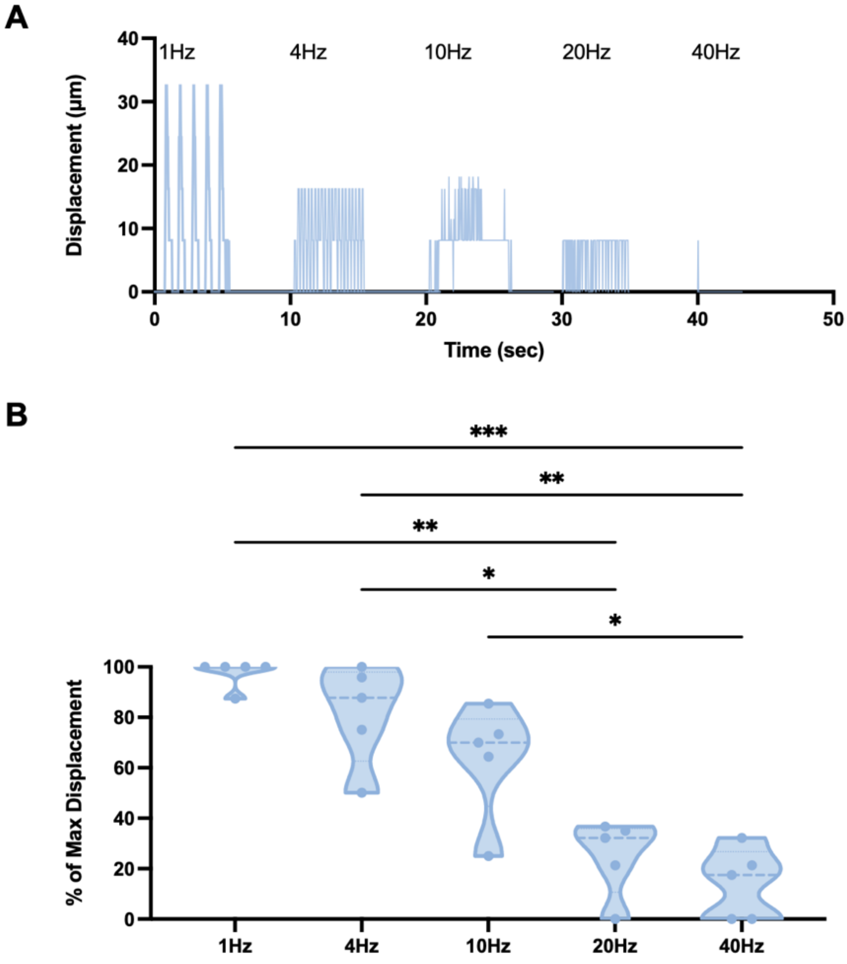

Figure S5. (**A**) Dynamic range of oscillation at peak contractile displacement for a representative MTU in response to stimulation at 1, 4, 10, 20, and 40 Hz. While low-frequency stimulation (1 Hz) captures the full range of muscle contractile stroke, oscillation amplitude

decreases at higher frequencies (4, 10, 20 Hz) and drops to zero at 40 Hz, indicating tetanic contraction. (**B**) Comparison of average contractile displacement for n = 5 MTUs at different frequencies, with each data point representing an average of 10 displacement measurements per MTU at that frequency. Values are represented as a percentage of maximum displacement observed for each MTU at 1 Hz stimulation. Brown-Forsythe and Welch ANOVA with Dunnett's multiple comparisons test. * $p < 0.05$, ** $p < 0.01$, *** $p < 0.0001$.

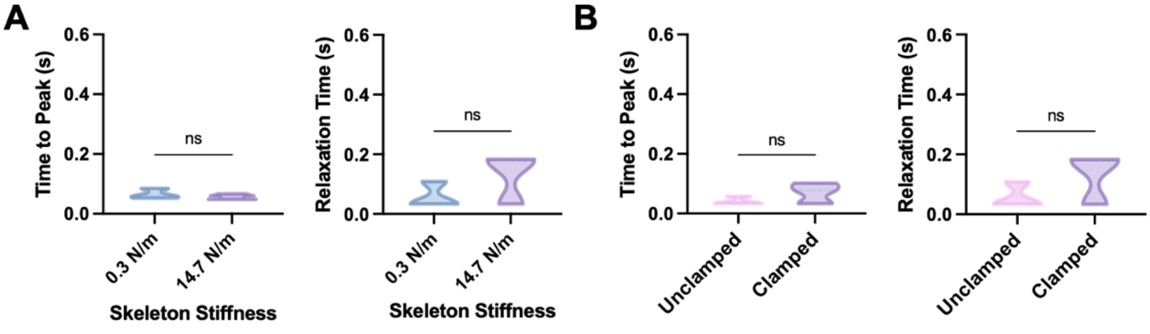

Figure S6. Contractile dynamics of MTUs. (**A**) Time-to-peak-force (left) and relaxation time (right) of MTUs on compliant flexure skeletons (0.3 N/m) and stiff gripper flexure skeletons (14.7 N/m). (**B**) Time-to-peak-force (left) and relaxation time (right) of MTUs on stiff gripper flexure skeletons (14.7 N/m) in unclamped and clamped configurations.

Video S1. Impact of MTU pre-stretch on contractile displacement.

Video S2. Impact of 1, 2, and 4 Hz stimulation frequency on MTU contractile displacement.

Video S3. Impact of 1, 4, 10, 20, and 40 Hz stimulation frequency on MTU contractile displacement.

Video S4. MTU fatigue over 30 minutes at 4 Hz stimulation.

Video S5. MTUs on stiff gripper skeletons.
